# Supplementary material for: Identification of photoperiod-induced specific miRNAs in the adrenal glands of Sunite sheep (Ovis aries)
Source: Front Vet Sci. 2022 Jul 22;9:888207. doi: 10.3389/fvets.2022.888207 (PMC9354845; doi:10.3389/fvets.2022.888207)
Supplement: Supplementary file 1 [file Data_Sheet_1.ZIP › Supplementary file/miRDeep2 file.zip!/miRDeep2 file/Novel_Structure/LP42A2/Novel_502.pdf]

Provisional ID	:	NC_019484.2_38372
Score total	:	1.6
Score for star read(s)	:	-1.3
Score for read counts	:	0
Score for mfe	:	1.3
Score for randfold	:	1.6
Score for cons. seed	:	
Total read count	:	168
Mature read count	:	168
Loop read count	:	0
Star read count	:	0

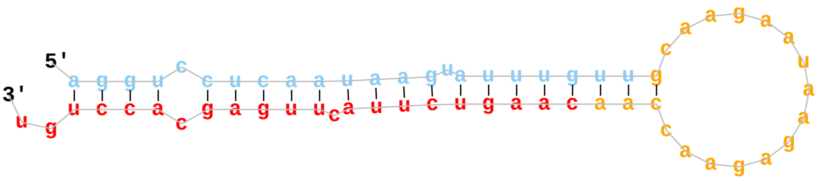

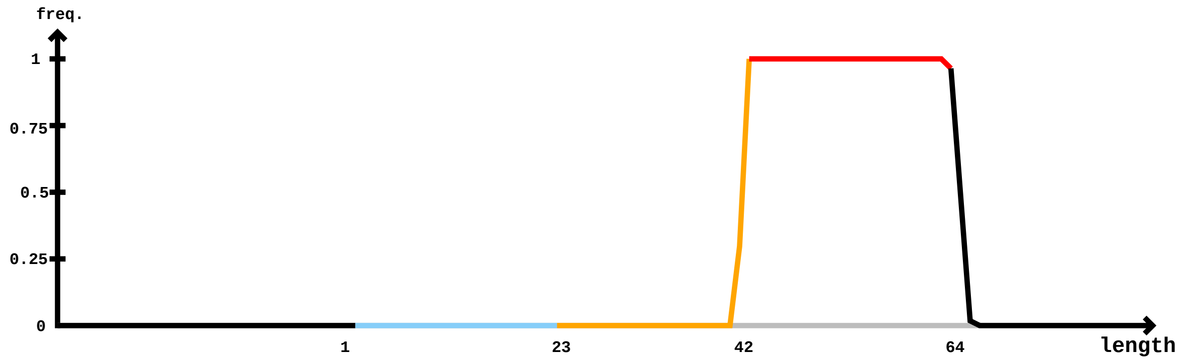

Star Mature

```
5'- auuguguuucuucaugccugacauauagcagguccucaauaaguauuuguugcaagaauaagagaaccaacaagucuuacuugagcaccuguuauguggaggauaaaaagcu   -3'    exp
    .....((((.((.((.(((..((((((((((((.(((((((((.((((((((...............)))))))))))).))))).)))))))))))).))))))).)))).    reads       mm          sample
    .....................................................................acaagucuuacuugagcaccug.....................    2           0           seq
    .....................................................................acaagucuuacuugagcaccugu....................    35          0           seq
    .....................................................................acaagucuuacuugagcaccuguu...................    13          0           seq
    ......................................................................caagucuuacuugagcaccug.....................    4           0           seq
    ......................................................................caagucuuacuugagcaccugu....................    94          0           seq
    ......................................................................caagucuuacuugagcaccuguu...................    17          0           seq
    ......................................................................caagucuuacuugagcaccuguua..................    3           0           seq
```
